# Microscopy-based *Saccharomyces cerevisiae* complementation model reveals functional conservation and redundancy of N-terminal acetyltransferases

**DOI:** 10.1038/srep31627

**Published:** 2016-08-24

**Authors:** Camilla Osberg, Henriette Aksnes, Sandra Ninzima, Michaël Marie, Thomas Arnesen

**Affiliations:** 1 Department of Molecular Biology, University of Bergen, N-5020 Bergen, Norway; 2 Department of Surgery, Haukeland University Hospital, N-5021 Bergen, Norway

## Abstract

N-terminal acetylation is a highly abundant protein modification catalyzed by N-terminal acetyltransferases (NATs) NatA-NatG. The *Saccharomyces cerevisiae* protein Arl3 depends on interaction with Sys1 for its localization to the Golgi and this targeting strictly requires NatC-mediated N-terminal acetylation of Arl3. We utilized the Arl3 acetylation-dependent localization phenotype as a model system for assessing the functional conservation and *in vivo* redundancy of several human NATs. The catalytic subunit of human NatC, hNaa30 (Mak3), restored Arl3 localization in the absence of yNaa30, but only in the presence of either yeast or human Naa35 subunit (Mak10). In contrast, hNaa35 was not able to replace its yeast orthologue without the co-expression of hNaa30, suggesting co-evolution of the two NatC subunits. The most recently discovered and organellar human NAT, NatF/Naa60, restored the Golgi localization of Arl3 in the absence of yNaa30. Interestingly, this was also true for hNaa60 lacking its membrane-binding domain whereas hNaa50 did not complement NatC function. This *in vivo* redundancy reflects NatC and NatF´s overlapping *in vitro* substrate specificities. The yeast model presented here provides a robust and rapid readout of NatC and NatF activity *in vivo*, and revealed evolutionary conservation of the NatC complex and redundancy between NatC and NatF.

N-terminal (Nt) acetylation is a prevalent protein modification catalyzed by N-terminal acetyltransferases (NATs). It involves the transfer of an acetyl moiety from acetyl-coenzyme A (Ac-CoA) to the α-amino group of the first amino acid residue of a polypeptide. This is mainly considered to be a co-translational event although evidence exist that Nt-acetylation also occurs post-translationally^1^,^2^.

In the yeast *S. cerevisiae* more than 50% of the soluble proteome is subjected to Nt-acetylation, whereas in humans this number exceeds 70% 3,4,5. There is an increasing number of substrates uncovered for which Nt-acetylation has significant functional roles, including global protein folding^6^, protein-protein interaction^7^, protein stabilization or degradation^8^,^9^,^10^ and subcellular protein targeting^11^,^12^,^13^.

Five NATs have been identified in yeast, NatA-NatE, all of which are conserved to multicellular eukaryotes, which additionally contain NatF^4^,^14^. NatG was recently identified in the plant kingdom^15^. NatA and NatD target Met-cleaved N-termini^3^,^16^,^17^,^18^ whereas NatB, NatC, NatE and NatF acetylate Met-starting N-termini^4^,^5^,^16^,^19^,^20^,^21^. NatG is found in chloroplasts of plant cells and its activity is towards Ala-, Met-, Ser-, and Thr-starting N-termini^15^. NatC, NatE and NatF display overlapping substrate specificities *in vitro,* targeting Met-hydrophobic and Met-amphipathic N-termini^4^,^22^,^23^, whereas their *in vivo* substrates have been proposed to be distinct^5^,^24^. For instance, NatF localizes to the cytosolic surface of the Golgi apparatus and specifically Nt-acetylates membrane proteins with their N-termini facing the cytosol^5^.

NatC is a complex consisting of the catalytic subunit Naa30 (Mak3) and the auxiliary subunits Naa35 (Mak10) and Naa38 (Mak31)^22^,^25^,^26^. In yeast, the individual deletions of NatC subunits cause growth defects on non-fermentable carbon sources (e.g. glycerol) at elevated temperatures^25^. NatC knockdown in human cells results in reduced cell viability and p53-dependent apoptosis^22^. One of the verified NatC substrates is the yeast ADP-ribosylation factor-like 3 (Arl3) protein whose interaction with Golgi-anchored Sys1 strictly depends on NatC-mediated Nt-acetylation. In the absence of either Naa30 or Naa35, Arl3 remains unmodified and as a consequence is unable to associate with the Golgi^12^,^13^.

Although NatC has been deemed evolutionarily conserved in several aspects - such as *in vitro* substrate specificity, ribosome association and subunit composition^22^,^27^ - its conservation in terms of *in vivo* protein function remains unknown. In this study, we applied the yeast Arl3 localization phenotype as an *in vivo* system to study the functional conservation between yeast and human NatC. We found that the human Naa30-Naa35 NatC complex could functionally complement yeast lacking NatC activity, as indicated by its ability to restore the punctate Golgi localization of Arl3. Moreover, the possible NatC-redundancy of NatE and NatF was challenged in the Arl3 complementation assay to enlighten a potential *in vivo* redundancy. The organelle associated human NatF/Naa60, but not NatE/Naa50, was able to restore normal Arl3 distribution pattern in yeast. Surprisingly, also a truncated cytosolic hNaa60 complemented the Arl3 localization phenotype in the absence of active NatC complex. These results enlightened a functional difference *in vivo* among NatC, NatE and NatF, which are known to have partially overlapping *in vitro* substrate specificities.

## Results and Discussion

### Functional conservation of Naa30 revealed by Arl3 localization complementation assay

**Figure 1:**
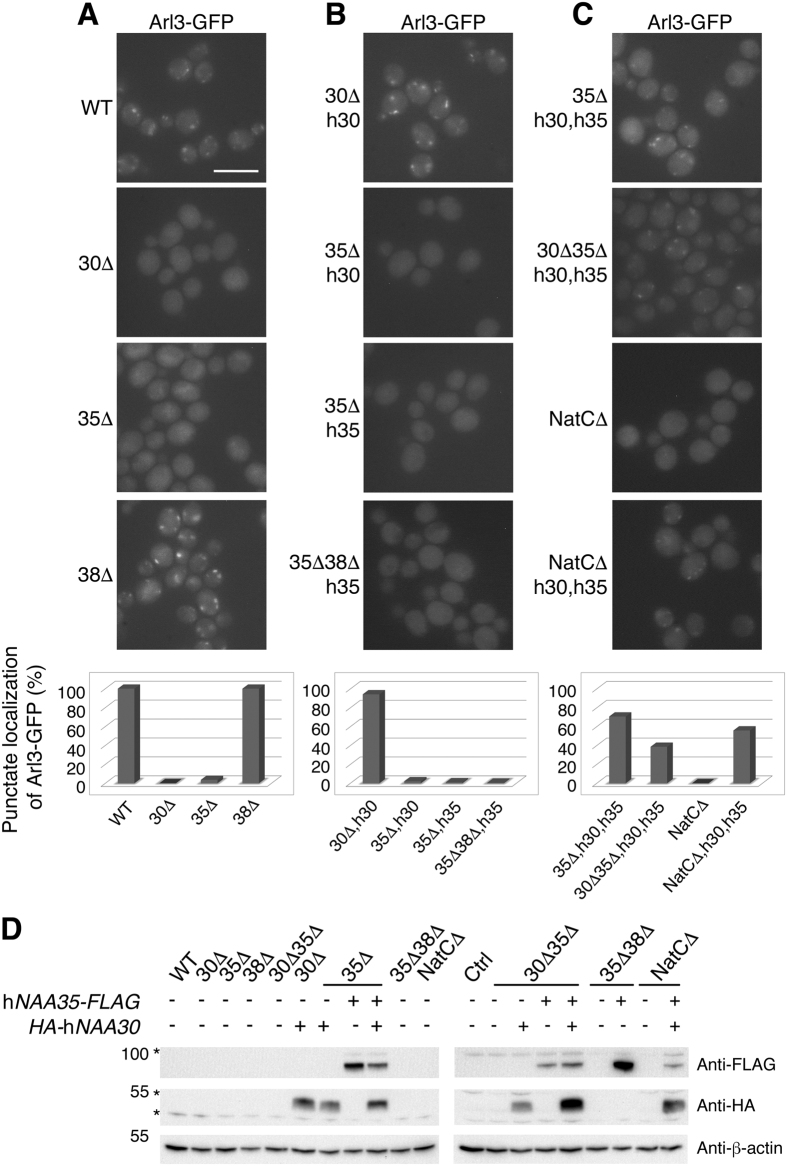
Arl3-GFP punctate localization as a model for active NatC revealed functional conservation of the NatC complex. (**A–C**) Live-cell microscopy of endogenous Arl3-GFP in yeast cells with the indicated genotypes. A corresponding bar graph below the fluorescent images shows the number of cells (n > 500 cells) harbouring a punctate localization pattern of Arl3-GFP. Scale bar, 5 μm. (**A**) WT, *naa30*Δ, *naa35*Δ and *naa38*Δ yeast. (**B**) Human Naa30 exogenously expressed in *naa30*Δ and *naa35*Δ cells, whereas hNaa35 was exogenously expressed in *naa35*Δ and *naa35*Δ*naa38*Δ yeast. (**C**) Exogenous expression of both human Naa30 and Naa35 in *naa35*Δ, *naa30*Δ*naa35*Δ and NatCΔ cells. (**D**) Western blot analysis using anti-HA and anti-FLAG antibody verified the exogenous expression of hNaa30 and hNaa35, respectively, in different yeast strains. Asterisks represent undetermined band detected by anti-FLAG or anti-HA (Sigma-Aldrich) antibodies. Abbreviations: 30Δ, *naa30*Δ; 35Δ, *naa35*Δ; 38Δ, *naa38*Δ; NatCΔ, *naa30*Δ*naa35*Δ*naa38*Δ; h30, HA-hNaa30; h35, hNaa35-FLAG.

The dependency of NatC-mediated Nt-acetylation of Arl3 for correct subcellular localization has previously been shown by fluorescence microscopy and mass spectrometry^12^,^13^. Here, we utilized the Arl3 localization as a model for NatC activity *in vivo* (Fig. 1). In WT cells, Arl3-GFP localized to the Golgi whereas in *naa30*Δ and *naa35*Δ cells the punctate localization was lost (Fig. 1A), as previously reported^12^,^13^. Yeast Naa38 was shown to be dispensable for Nt-acetylation of Arl3 by mass spectrometry^12^ and we accordingly observed the subcellular localization of *naa38*Δ yeast to be similar to the WT (Fig. 1A).

Next, we used the Arl3 localization model in various rescue assays and found the human orthologue of yNaa30 to be functionally conserved, as it was able to restore the mislocalization of Arl3-GFP in *naa30*Δ yeast (Fig. 1B). Previously, hNaa30 was reported to restore subcellular localization of Imh1, downstream of Arl3 function^13^. To investigate whether the complementation ability of hNaa30 was due to the catalytic subunit performing Nt-acetylation on its own or in a human-yeast interspecies complex with the other essential NatC subunit, yNaa35, we expressed HA-hNaa30 (hereafter referred to as hNaa30) in *naa35*Δ cells. In this yeast background, exogenously expressed hNaa30 failed to restore Arl3 localization (Fig. 1B), thus suggesting interspecies NatC functionality of hNaa30-yNaa35.

### Human Naa35 is unable to complement yeast Naa35 for Arl3 Nt-acetylation

**Figure 2:**
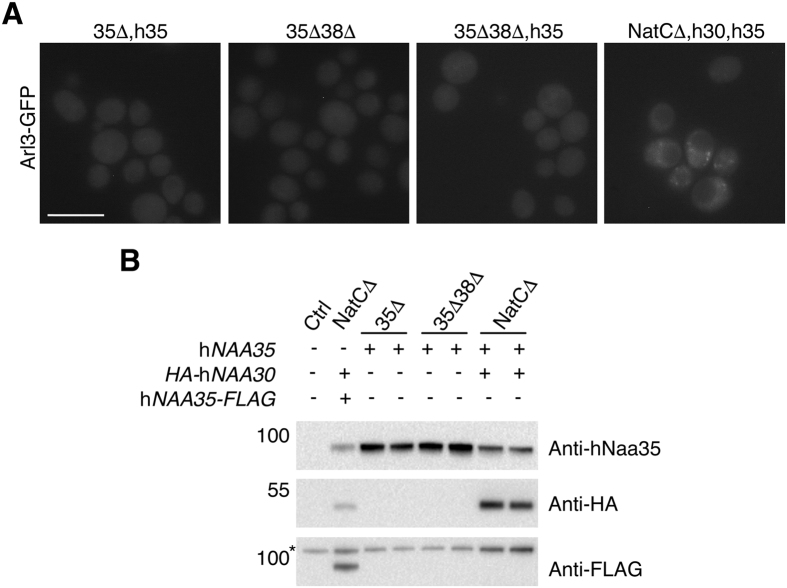
Untagged human Naa35 behaved similarly to hNaa35-FLAG. (**A**) Fluorescence microscopy of Arl3-GFP in different yeast strains. Untagged hNaa35 was exogenously expressed in *naa35*Δ and *naa35*Δ*naa38*Δ cells as well as together with hNaa30 in NatCΔ yeast. The Arl3 mislocalization phenotype was demonstrated in *naa35*Δ*naa38*Δ cells. Scale bar, 5 μm. (**B**) Western blot analysis of cell lysates revealed the presence of hNaa30 and hNaa35 (untagged) in the three yeast strains, represented by two individual clones. Asterisk indicates an undetermined band achieved by anti-FLAG. Abbreviations: 35Δ, *naa35*Δ; 38Δ, *naa38*Δ; NatCΔ, *naa30*Δ*naa35*Δ*naa38*Δ; h30, HA-hNaa30; h35, hNaa35.

The functional conservation of hNaa35 was then addressed by expressing hNaa35-FLAG (hereafter referred to as hNaa35) in *naa35*Δ cells, but this did not restore Arl3 localization. To exclude the possibility of yNaa38 hindering a necessary binding between hNaa35 and yNaa30 we further expressed hNaa35 in *naa35*Δ*naa38*Δ yeast. No rescue of Arl3 localization was seen in this condition either (Fig. 1B). Additionally, we performed the complementation assay using hNaa35 without the C-terminal FLAG-tag, which was also negative in terms of restoring Arl3 punctate localization (Fig. 2).

Taken together, these data demonstrate that hNaa30, but not hNaa35, can replace the corresponding yeast orthologue. Whereas hNaa30 functions in the presence of yNaa35, hNaa35 cannot function despite the presence of yNaa30, thus suggesting the formation of a functional hNaa30-yNaa35 interspecies complex, but not yNaa30-hNaa35.

### Human Naa30 and Naa35 cooperate to perform NatC activity in yeast

Based on the above results showing that co-expression of hNaa30 and yNaa35 restore NatC function and the inability of yNaa30 and hNaa35 to do so, we proceeded by studying both human NatC subunits in yeast. Human Naa30 and Naa35 were exogenously co-expressed in three different yeast strains: *naa35*Δ, *naa30*Δ*naa35*Δ, and *naa30*Δ*naa35*Δ*naa38*Δ (NatCΔ) (Fig. 1D). For all deletion strains, a clear rescue of Arl3 localization was observed, indicating that NatC activity was present (Fig. 1C). Thus, the two human subunits Naa30 and Naa35 cooperated and performed NatC activity on a yeast substrate protein *in vivo*. From this, we suggest a functional conservation of the Naa30-Naa35 NatC complex between *S. cerevisiae* and humans, and that there has been a co-evolution of these subunits since heterologous expression of yNaa30 and hNaa35 is non-functional. Our observation that human Naa30-Naa35 NatC complex is functionally active in yeast and complements yNatC, is analogous to earlier complementation studies of NatA and NatB^3^,^21^, and suggests a large degree of conservation of these major NATs from yeast to humans. However, the yeast/human Naa30 complementation observed here, differs from those of NatA and NatB studies in which the human catalytic subunits alone (Naa10 and Naa20, respectively) cannot phenotypically complement a yeast strain lacking its yeast orthologue^3^,^21^.

### The punctate localization pattern of Arl3 corresponds to Golgi structures

**Figure 3:**
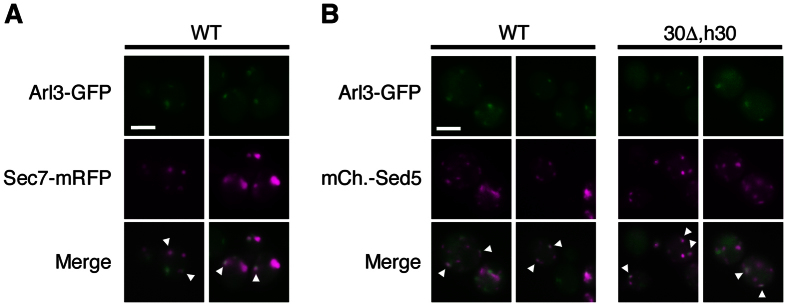
Partial co-localization was observed between Arl3 and two individual Golgi marker proteins. (**A**) Imaging of live WT yeast expressing endogenous Arl3-GFP and exogenous Sec7-mRFP. (**B**) Endogenous Arl3-GFP and exogenous mCherry-Sed5 were imaged in live WT cells and in *naa30*Δ cells exogenously expressing HA-hNaa30. Scale bars, 2 μm. Abbreviations: 30Δ, *naa30*Δ; h30, HA-hNaa30.

To verify that the observed localization pattern of Arl3 in the rescue assays did indeed represent restoration of the Arl3 WT localization, the *trans*-Golgi Sec7-mRFP and *cis*-Golgi mCherry-Sed5 marker proteins were individually expressed in Arl3-GFP cells. Both Golgi markers partially co-localized with Arl3-GFP in the WT, apparently with equal amount of overlap (Fig. 3). We further examined the restored punctate Arl3-GFP localization in *naa30*Δ cells expressing hNaa30 and detected similar co-localization with mCherry-Sed5 as for the WT yeast (Fig. 3B). This confirmed that the reappeared punctate structures detected represents restored Arl3 Golgi localization.

### Overlapping *in vivo* activities of human NatC and NatF in yeast

**Figure 4:**
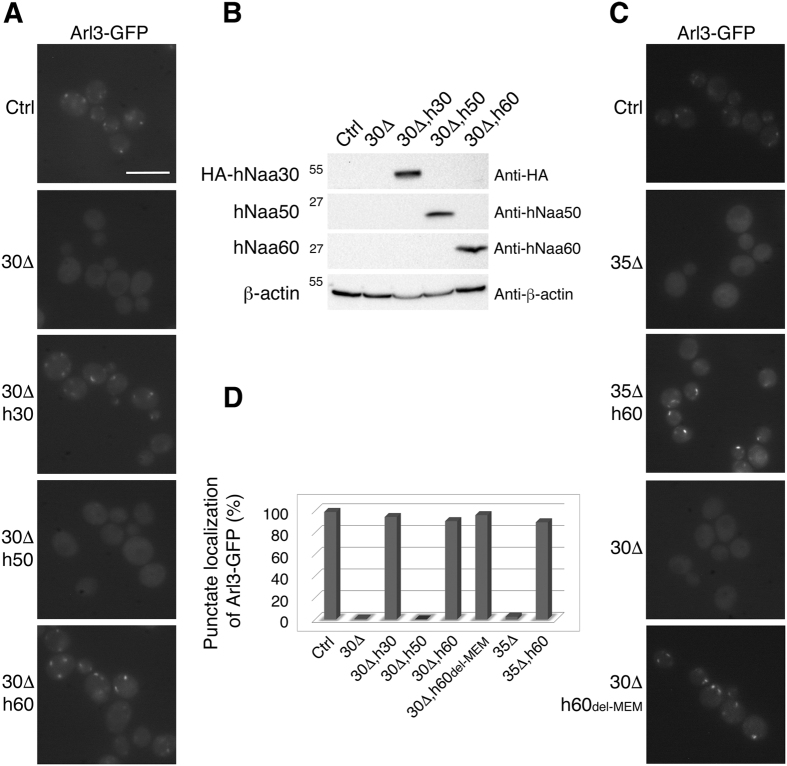
Human Naa60 has *in vivo* redundancy with Naa30 that is independent of its organellar localization. (**A**) Live-cell microscopy of Arl3-GFP in yeast genetically modified as indicated. Restoration of Arl3-GFP localization by hNaa30 is shown as a positive control. (**A**,**C**) Scale bar, 5 μm. (**B**) Western blot analysis of cell lysates verified the expression of exogenous human Naa30, Naa50 or Naa60 in yeast strains shown in (**A**). (**C)** Detection of endogenous Arl3-GFP in live yeast. Human Naa60 and a C-terminally truncated variant Naa60_1−184_ (h60del-MEM) were exogenously expressed in *naa35*Δ and *naa30*Δ cells, respectively. (**D**) Amount of cells for which the Arl3-GFP punctate localization was observed in the indicated yeast strains. The number from *naa30*Δ yeast expressing hNaa30 is a re-representation of data in Fig. 1B as a positive control reference (n > 500 cells). Abbreviations: 30Δ, *naa30*Δ; 35Δ, *naa35*Δ; h30, HA-hNaa30; h50, hNaa50; h60, hNaa60; h60del-MEM, hNaa60 without membrane binding region.

Human NatC, NatE and NatF display overlapping *in vitro* substrate specificities^4^,^22^,^23^. Furthermore, human NatE and NatF exhibit overlapping *in vivo* substrate profiles when expressed in yeast^4^,^24^. All three enzymes recognize Met-starting N-termini containing a hydrophobic or amphipathic amino acid residue in the second position. Arl3 has a Met-Phe-starting N-terminus and thus may be a putative target of these enzymes. To challenge the suggested substrate redundancy between human NatC, NatE and NatF *in vivo*, we again utilized the Arl3 complementation model (Fig. 4). Here, the human catalytic subunits Naa30, Naa50 and Naa60 were individually expressed in Arl3-GFP *naa30*Δ cells. Human Naa50, which in human cells is cytosolic and partially ribosome-associated^23^,^28^, was not able to complement yNaa30 whereas hNaa60 almost completely reinstated the punctate Arl3 localization, similar to hNaa30 (Fig. 4A). The same was true for hNaa60 expressed in *naa35*Δ cells (Fig. 4C), indicating that hNaa60 may operate independently of any of the yNatC subunits, unlike hNaa30, which requires the assumed ribosome binding subunit Naa35.

**Figure 5:**
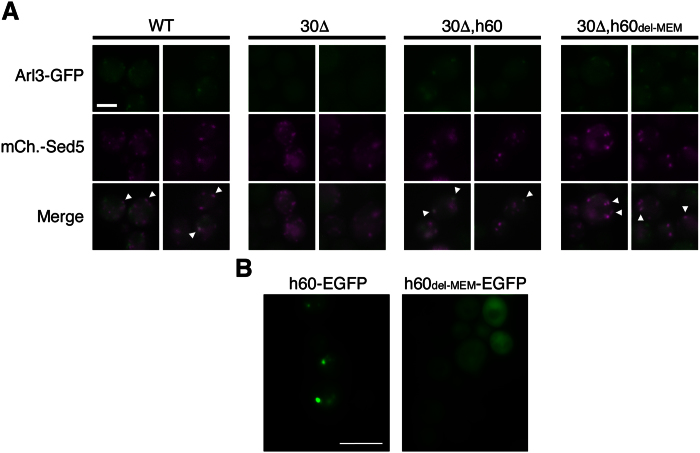
Human Naa60-rescued Arl3 partially co-localizes with the *cis*-Golgi marker Sed5. Yeast cells of the indicated genotypes were imaged for the detection of endogenous Arl3-GFP and exogenous mCherry-Sed5. Scale bar, 2 μm. (**B**) Subcellular localization of C-terminally EGFP-tagged hNaa60 and hNaa60_del-MEM_ in live yeast cells. Scale bar, 5 μm. Abbreviations: 30Δ, *naa30*Δ; h60, hNaa60; h60del-MEM, hNaa60 without membrane binding region.

Naa60 was recently described in human cells as the first organellar NAT and its catalytic activity is facing towards the cytosol^5^. It might be possible that the ability of human Naa60 to complement the NatC activity on Arl3 relates to Naa60´s association with the yeast Golgi membrane. However, upon expression of truncated Naa60_del-MEM_, without the C-terminal end that mediates Naa60 membrane-association in mammalian cells, (ref. 5), the Arl3 localization pattern was again represented as punctate structures that partially co-localized with the *cis*-Golgi marker Sed5 (Figs 4C and 5A). Human Naa60 and Naa60_del-MEM_ fused to a C-terminal EGFP tag mainly localized to punctate structures and the cytosol, respectively (Fig. 5B). Taken together these data may indicate a post-translational activity of NatF/Naa60 towards Arl3-GFP at the Golgi or freely in the cytosol. In any case, the data verify Met-Phe as an *in vivo* N-terminal target sequence of NatF and demonstrate the ability of human Naa30 and Naa60 to act on the same substrate *in vivo*^4^.

From these results, it is clear that human Naa60 and Naa60_del-MEM_, but not hNaa50, are able to rescue the Arl3 localization phenotype, thus enlightening an interesting Nt-acetylation performance of NatF as well as functional redundancy with NatC. Moreover, human Naa50 acts as a negative control in the assay for Arl3 localization, excluding mere NAT overexpression to be sufficient for phenotype rescue.

## Conclusions

**Figure 6:**
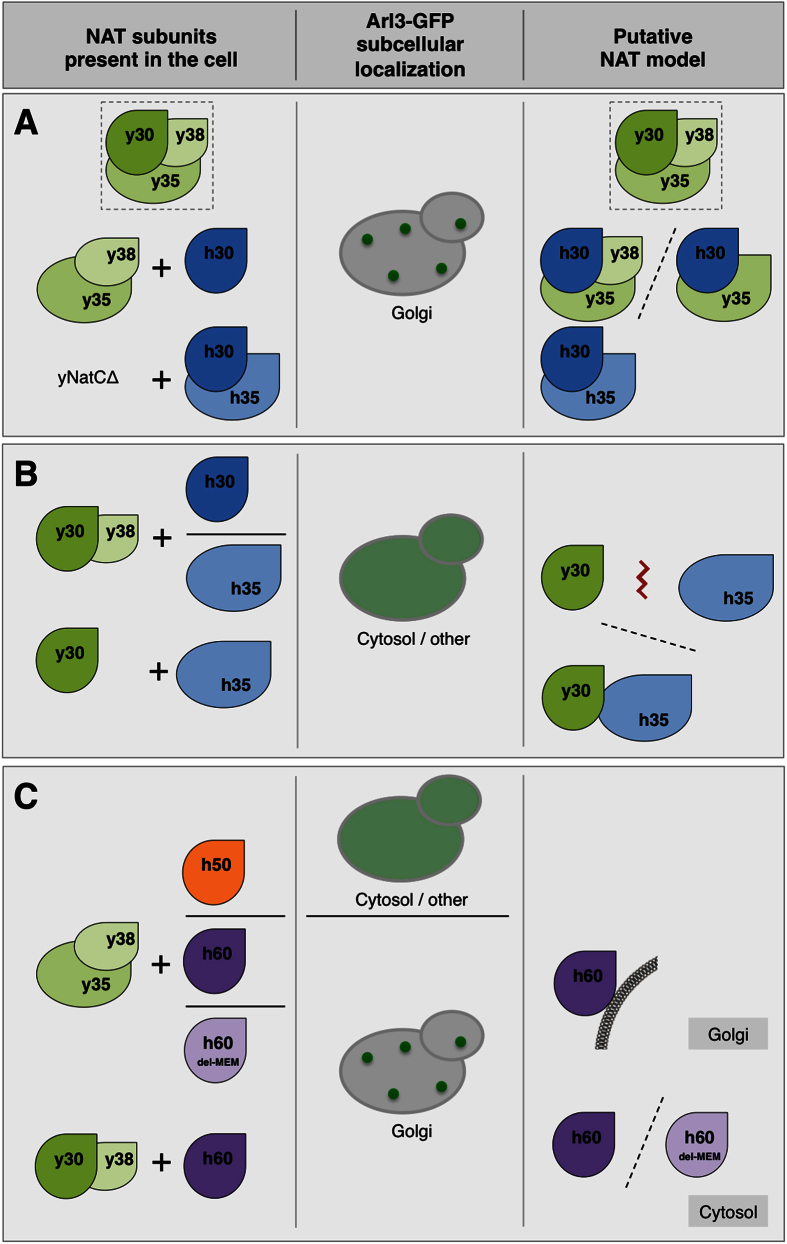
Putative model of the NatC, NatE and NatF subunits and their effect on Arl3 localization. In WT yeast cells Arl3 is N-terminally acetylated by the NatC complex (Naa30 and Naa35, whereas Naa38 is dispensable for Arl3-acetylation) and targeted to the Golgi transmembrane protein Sys1, mediating Arl3 punctate Golgi localization. However, in the absence of a functional NatC complex Arl3 remains unacetylated after protein synthesis and the targeting is abrogated, leading to Arl3 cytosolic mislocalization^12^,^13^. (**A**) Human Naa30 is able to function in complex with yNaa35 (and possibly yNaa38) or hNaa35, indicating a functionally conserved catalytic subunit between yeast and humans. WT yNatC is depicted framed in dotted line. (**B**) No Nt-acetylation activity of hNaa30 is observed in the absence of yeast or human Naa35, which is thought to be the ribosome-binding subunit. Human Naa35 cannot replace its yeast orthologue and function together with yNaa30, neither in the presence nor absence of yNaa38. (**C**) Naa30 and Naa60 exhibit *in vivo* redundancy. The Arl3 localization phenotype is complemented by human Naa60 and Naa60_del-MEM_ in both *naa30*Δ and *naa35*Δ yeast. Based on the subcellular localizations of human Naa60 and Naa60_del-MEM_, a possible post-translational NAT activity at the cytosolic side of Golgi or freely in the cytosol is suggested. Abbreviations: y30, yNaa30; y35, yNaa35; y38, yNaa38; h30, hNaa30; h35, hNaa35; h50, hNaa50; h60, hNaa60; h60del-MEM, hNaa60 without membrane binding region; Ac-CoA, acetyl-coenzyme A.

We applied *S. cerevisiae* as a model system to study the *in vivo* function of human NATs. The NatC-dependent subcellular localization of yeast Arl3 proved to be a suitable model for studying functional conservation among NatC orthologues as well as diverging *in vivo* function among NatC, NatE and NatF harbouring overlapping *in vitro* substrate specificities (Fig. 6). Naa30 was defined as functionally conserved between yeast and humans through its ability to complement Arl3 mislocalization, which interestingly required the presence of either yeast or human Naa35; accordingly we propose that NatC complex formation occurred (Fig. 6A). Human Naa35 on the other hand, could not complement its yeast orthologue and we suggest this to be due to insufficient binding between yNaa30 and hNaa35 or formation of an impaired complex (Fig. 6B). Taken together, these results indicate that the two subunits Naa30 and Naa35 have co-evolved.

Despite human Naa30, Naa50 and Naa60’s shared catalytic activity towards specific N-terminal sequences, we found opposite complementation patterns of NatE/Naa50 and NatF/Naa60 for the NatC-deficient phenotype of Arl3. This clearly hints to unique *in vivo* functions of the two. Furthermore, in light of the different subcellular localizations of Naa60 and Naa60_del-MEM_ in human cells^5^ and in yeast cells, both being able to rescue the Arl3 localization (Fig. 6C), the difference between Naa50 and Naa60 may reflect differences in the substrate binding site rather than differences in subcellular localization. This assumption is also based on the crystal structures and catalytic efficiencies of human Naa50^29^ and Naa60^30^ suggesting that Naa60 may accommodate larger and more bulky substrate side chains as compared to Naa50.

The yeast model presented here may act as a useful and rapid *in vivo* setup to assess the functionality of various NAT subunits, meaning isoforms of human Naa30, Naa35 and Naa60. For a Naa10 mutant causing the Ogden syndrome^31^,^32^, a yeast model provided useful insights into the impaired functionality of NatA^33^ Similarly, the yeast model described here may be used in any future studies of pathological mutants of NatC or NatF.

## Methods

### Yeast Cultivation

The yeast strains used in this study (see Supplementary Table S1) are derived from one of the two parental strains BY4741 (*MATa his3Δ1 leu2Δ0 met15Δ0 ura3Δ0*) and BY4742 (*MATα his3Δ1 leu2Δ0 lys2Δ0 ura3Δ0*). According to their genotype and required selection, the cells were either grown in YPD (Sigma-Aldrich, YPD Broth #Y1375 and YPD Agar #Y1500) or appropriate synthetic defined dropout media (Sunrise Science Products, SD-URA #1703-500, SD-LEU #1707-500, SD-LEU-URA #1721-500, Agar #1910-500). Protein expression of mCherry-Sed5 under the control of the MET25 promoter required growth medium with maximum 10 μg/ml methionine.

### Plasmid construction

A record of all plasmids and primers used is found in Supplementary Table S2 and Table S3, respectively. Human *NAA30* (Gene ID 122830) was sub-cloned from pRS315-*HA-hMAK3*, which was a kind gift from Professor Sean Munro, MRC, Cambridge^13^, into the pBEVY-U yeast vector^34^ downstream of the *ADH1* promoter using restriction enzyme sites XmaI (5´) and EcoRI (3´). The N-terminal HA-tag was retained from the original gene construct.

Human *NAA35* (Gene ID 60560) was amplified from pCMV6-AC-*NAA35-GFP* (OriGene, #RG213022) with the insertion of XbaI (5´) and PstI (3´) restriction enzyme sites as well as a C-terminal FLAG-tag and ligated into pBEVY-L after the *GPD* promoter. Additionally, pBEVY-L-h*NAA35* was generated by introducing a stop codon downstream of the *NAA35* ORF by mutagenesis (Agilent Technologies, #210514).

A plasmid containing human Naa60_1-184_ (Naa60_del-MEM_) was generated by mutagenesis of pBEVY-U-*hNAA60* 4, introducing a stop codon after the codon representing amino acid 184 of Naa60. Moreover, pBEVY-U-*hNAA60-EGFP* and pBEVY-U-*hNAA60*_*1−182*_*-EGFP* (Naa60_del-MEM_) were generated for subcellular localization studies by subcloning from mammalian pEGFP-N1 vectors in which the respective *NAA60* sequences were inserted between NheI and KpnI sites. The C-terminally EGFP-tagged *NAA60* constructs were inserted between XbaI (5´) and SalI (3´) restriction enzymes sites in the pBEVY-U vector after the *GPD* promoter.

Plasmid p415*MET25-mRFP-SED5* was kindly provided by Professor Blanche Schwappach, UMG, Göttingen^35^ and pRS316-*SEC7-mRFP* was from RIKEN BRC DNA BANK (#RDB08663)^36^.

### Yeast transformation

Yeast transformation was based on a method developed by Gietz and Schiestl^37^. Exponentially growing yeast cells (10 ml culture, OD_600_ 1.0) were harvested, washed twice with water and resuspended in 240 μl 50% PEG-3350 before addition of 36 μl 1 M lithium acetate, 100 μg salmon sperm ssDNA, DNA template (10 μg gene deletion cassette or 5 μg plasmid), and water up to 360 μl. After a 40 min heat-shock at 42 °C the cells were washed four times with water, before plating or incubation in YPD, depending on selection.

Yeast gene deletions were performed by homologous recombination and a complete overview of the primers used to amplify the respective deletion cassettes as well as the PCR-based screen for positive mutants are listed in Supplementary Table S3.

### Protein extraction and Western blot analysis

Protein extraction was done according to Kushnirov^38^ with modifications. Exponentially growing yeast cells (4 ml yeast culture, OD_600_ 1.0) were harvested for 10 min at 5,000 × *g*. The pellet was washed twice with water, resuspended in 200 μl water before adding 200 μl 0.2 M NaOH for 5 min. Cells were harvested for 1 min at 12,000 × *g*, resuspended and boiled for 3 min in 100 μl 1x Sample buffer (10% glycerol, 2% SDS, 50 mM Tris-HCl, pH 6.8, 0.0025% bromophenol blue, 50 mM dithiothreitol), before pelleting cell debris. Typically, 6 μl supernatant was loaded for SDS-PAGE on 4–20% Mini-PROTEAN^®^ gels (Bio-Rad, #456–8093).

Exogenous expression of HA-hNaa30 and hNaa35-FLAG were verified by Western blotting using anti-HA (1:400, rabbit (Rb), polyclonal, Sigma-Aldrich, #H6908) or anti-HA (1:1000, Rb, polyclonal, Abcam, #ab9110) and anti-FLAG (1:1000, Ms, M2 monoclonal, Sigma-Aldrich, #F1804) or anti-hNaa35 (1:1000, Rb, polyclonal, Sigma-Aldrich, #HPA021547), respectively. Beta-actin served as loading control and was detected by anti-β-actin (1:4000, Ms, monoclonal, Abcam, #8224). Additionally, protein expression of human Naa50 and Naa60 were verified by anti-hNaa50 (1:500, Rb, polyclonal, custom made raised against a peptide representing the amino acids 150–163 of hNaa50, BioGenes) and anti-hNaa60 (1:500, Rb, polyclonal, custom made raised against a protein representing the amino acids 1–184 of hNaa60, BioGenes), respectively.

### Fluorescence microscopy

Cells in exponential growth phase (OD_600_ 0.8–1.2) were pelleted and washed three times in 1x PBS, 2% glucose. Fluorescence images were acquired in the washing solution on a Leica DMI6000 B widefield microscope as previously described^39^. Recorded images were processed using Photoshop CS5 (Adobe Systems, San Jose, CA, USA). Arl3-GFP subcellular localization was characterized as normal (punctate) or mislocalization (other). More than 500 cells were counted per strain.

## Additional Information

**How to cite this article**: Osberg, C. *et al.* Microscopy-based *Saccharomyces cerevisiae* complementation model reveals functional conservation and redundancy of N-terminal acetyltransferases. *Sci. Rep.* **6**, 31627; doi: 10.1038/srep31627 (2016).

## Supplementary Material

Supplementary Information
