# Supplementary material for: Microscopy-based Saccharomyces cerevisiae complementation model reveals functional conservation and redundancy of N-terminal acetyltransferases
Source: Sci Rep. 2016 Aug 24;6:31627. doi: 10.1038/srep31627 (PMC4995432; doi:10.1038/srep31627)
Supplement: Supplementary Information [file srep31627-s1.pdf]

Supplementary Information

Title: Microscopy-based *Saccharomyces cerevisiae* complementation model reveals functional conservation and redundancy of N-terminal acetyltransferases

Authors: Camilla Osberg[1,2], Henriette Aksnes[1], Sandra Ninzima[1], Michaël Marie[1], Thomas Arnesen[1,2*]

Supplementary tables

Table S1. Yeast strains of this study.

yTA #	Short name	Genotype	pTA #	Use	Source
36	WT	BY4742		SG	EUROSCARF (#Y10000)
44	30Δ	BY4742; *naa30Δ::kanMX4*		SG	EUROSCARF (#Y15470)
59	WT	BY4741		SG	EUROSCARF (#Y00000)
256	Arl3-GFP, WT	BY4741; *ARL3::GFP-HIS3MX6*		Fig. 1A,D, SG	Invitrogen (GFP-collect.)
266	Arl3-GFP, 30Δ	BY4741; *ARL3::GFP-HIS3MX6, naa30Δ::kanMX4*		Fig. 1A,D, SG	[1]
480	Arl3-GFP, 35Δ	BY4741; *ARL3::GFP-HIS3MX6; naa35Δ::kanMX4*		Fig. 1A,D, SG	This study
485	Arl3-GFP, 38Δ	BY4741; *ARL3::GFP-HIS3MX6; naa38Δ::kanMX4*		Fig. 1A,D	This study
375	Arl3-GFP, 30Δ, h30	BY4741; *ARL3::GFP-HIS3MX6*; *naa30Δ::kanMX4*; [*pBEVY-U-HA-hNAA30*]	231	Fig. 1B,D, Fig. 4	This study
728	Arl3-GFP, 35Δ, h30	BY4741; *ARL3::GFP-HIS3MX6; naa35Δ::kanMX4; [pBEVY-U-HA-hNAA30]*	231	Fig. 1B,D	This study
730	Arl3-GFP, 35Δ, h35	BY4741; *ARL3::GFP-HIS3MX6; naa35Δ::kanMX4; [pBEVY-L-hNAA35-FLAG]*	226	Fig. 1B,D	This study
772	Arl3-GFP, 35Δ38Δ, h35	BY4741; *ARL3::GFP-HIS3MX6; naa35Δ::kanMX4; naa38Δ::hphNT1; [pBEVY-L-hNAA35]*	226	Fig. 1B,D	This study
732	Arl3-GFP, 35Δ, h30, h35	BY4741; *ARL3::GFP-HIS3MX6; naa35Δ::kanMX4; [pBEVY-U-HA-hNAA30]; [pBEVY-L-hNAA35-FLAG]*	226, 231	Fig. 1C,D	This study
698	Arl3-GFP, 30Δ35Δ, h30, h35	BY4741; *ARL3::GFP-HIS3MX6; naa30Δ::kanMX4; naa35Δ::natNT2; [pBEVY-U-HA-hNAA30]; [pBEVY-L-hNAA35]*	226, 231	Fig. 1C,D	This study

yTA #	Short name	Genotype	pTA #	Use	Source
751	Arl3-GFP, NatCΔ, pBEVY-U, pBEVY-L	BY4741; *ARL3::GFP-HIS3MX6; naa30Δ::kanMX4; naa35Δ::natNT2; naa38Δ::hphNT1; [pBEVY-U]; [pBEVY-L]*	53, 224	Fig. 1C,D	This study
753	Arl3-GFP, NatCΔ, h30, h35	BY4741; *ARL3::GFP-HIS3MX6; naa30Δ::kanMX4; naa35Δ::natNT2; naa38Δ::hphNT1; [pBEVY-U-HA-hNAA30]; [pBEVY-L-hNAA35-FLAG]*	226, 231	Fig. 1C,D	This study
692	Arl3-GFP, 30Δ35Δ, pBEVY-U, pBEVY-L	BY4741; *ARL3::GFP-HIS3MX6; naa30Δ::kanMX4; naa35Δ::natNT2; [pBEVY-U]; [pBEVY-L]*	53, 224	Fig. 1D	This study
696	Arl3-GFP, 30Δ35Δ, h30, pBEVY-L	BY4741; *ARL3::GFP-HIS3MX6; naa30Δ::kanMX4; naa35Δ::natNT2; [pBEVY-U-HA-hNAA30]; [pBEVY-L]*	224, 231	Fig. 1D	This study
694	Arl3-GFP, 30Δ35Δ, pBEVY-U, h35	BY4741; *ARL3::GFP-HIS3MX6; naa30Δ::kanMX4; naa35Δ::natNT2; [pBEVY-U]; [pBEVY-L-hNAA35]*	53, 226	Fig. 1D	This study
673	Arl3-GFP, pBEVY-L, pBEVY-U	BY4741; *ARL3::GFP-HIS3MX6; [pBEVY-L]; [pBEVY-U]*	53, 224	Fig. 1D (Ctrl)	This study
770	Arl3-GFP, 35Δ38Δ, pBEVY-L	BY4741; *ARL3::GFP-HIS3MX6; naa35Δ::kanMX4; naa38Δ::hphNT1; [pBEVY-L]*	224	Fig. 2	This study
858	Arl3-GFP, 35Δ, h35 (n.t.)	BY4741; *ARL3::GFP-HIS3MX6; naa35Δ::kanMX4; [pBEVY-L-hNAA35]*	342	Fig. 2	This study
860	Arl3-GFP, 35Δ38Δ, h35 (n.t.)	BY4741; *ARL3::GFP-HIS3MX6, naa35Δ::kanMX4; naa38Δ::hphNT1; [pBEVY-L-hNAA35]*	342	Fig. 2	This study
862	Arl3-GFP, NatCΔ, h30, h35 (n.t.)	BY4741; *ARL3::GFP-HIS3MX6; naa30Δ::kanMX4; naa35Δ::natNT2; naa38Δ::hphNT1; [pBEVY-U-HA-hNAA30]; [pBEVY-L-hNAA35]*	231, 342	Fig. 2	This study
771	Arl3-GFP, 35Δ38Δ, pBEVY-L	BY4741; *ARL3::GFP-HIS3MX6; naa35Δ::kanMX4; naa38Δ::hphNT1; [pBEVY-L]*; clone 2	224	Fig. 2B	This study
859	Arl3-GFP, 35Δ, h35 (n.t.)	BY4741; *ARL3::GFP-HIS3MX6; naa35Δ::kanMX4; [pBEVY-L-hNAA35]*; clone 2	342	Fig. 2B	This study

yTA #	Short name	Genotype	pTA #	Use	Source
861	Arl3-GFP, 35Δ38Δ, h35 (n.t.)	BY4741; *ARL3::GFP-HIS3MX6, naa35Δ::kanMX4; naa38Δ::hphNT1; [pBEVY-L-hNAA35]*; clone 2	342	Fig. 2B	This study
863	Arl3-GFP, NatCΔ, h30, h35 (n.t.)	BY4741; *ARL3::GFP-HIS3MX6; naa30Δ::kanMX4; naa35Δ::natNT2; naa38Δ::hphNT1; [pBEVY-U-HA-hNAA30]; [pBEVY-L-hNAA35]*; clone 2	231, 342	Fig. 2B	This study
855	Arl3-GFP, Sec7-mRFP	BY4741; *ARL3::GFP-HIS3MX6; [*316*-Sec7-mRFP]*	321	Fig. 3A	This study
830	Arl3-GFP, mCherry-Sed5	BY4741; *ARL3::GFP-HIS3MX6; [p415Met25-mCherry-Sed5]*	340	Fig. 3B	This study
832	Arl3-GFP, 30Δ, h30, mCherry-Sed5	BY4741; *ARL3::GFP-HIS3MX6; naa30Δ::kanMX4*; [*pBEVY-U-HA-hNAA30*]; *[p415Met25-mCherry-Sed5]*	231, 340	Fig. 3B	This study
371	Arl3-GFP, pBEVY-U	BY4741; *ARL3::GFP-HIS3MX6; [pBEVY-U]*	53	Fig. 4	This study
373	Arl3-GFP, 30Δ, pBEVY-U	BY4741; *ARL3::GFP-HIS3MX6; naa30Δ::kanMX4; [pBEVY-U]*	53	Fig. 4	This study
377	Arl3-GFP, 30Δ, h50	BY4741; *ARL3::GFP-HIS3MX6; naa30Δ::kanMX4; [pBEVY-U-hNA50]*	285	Fig. 4A,B,D	This study
379	Arl3-GFP, 30Δ, h60	BY4741; *ARL3::GFP-HIS3MX6; naa30Δ::kanMX4; [pBEVY-U-hNAA60]*	286	Fig. 4A,B,D	This study
954	Arl3-GFP, 30Δ, $h60_{1\text{-}184}$	BY4741; *ARL3::GFP-HIS3MX6; naa30Δ::kanMX4; [pBEVY-U-hNAA60*$_{1\text{-}184}$*]*	378	Fig. 4C,D, SG	This study
509	Arl3-GFP, 35Δ, pBEVY-U	BY4741; *ARL3::GFP-HIS3MX6; naa35Δ::kanMX4; [pBEVY-U]*	53	Fig. 4C,D	This study
532	Arl3-GFP, 35Δ, h60	BY4741; *ARL3::GFP-HIS3MX6; naa35Δ::kanMX4; [pBEVY-U-hNAA60]*	286	Fig. 4C,D	This study
1010	Arl3-GFP, pBEVY-U, mCherry-Sed5	BY4741; *[pBEVY-U]; [p415Met25-mCherry-Sed5]*	53, 340	Fig. 5A	This study
1012	Arl3-GFP, 30Δ, mCherry-Sed5	BY4741; *ARL3::GFP-HIS3MX6; naa30Δ::kanMX4*; [*pBEVY-U]; [p415Met25-mCherry-Sed5]*	53, 340	Fig. 5A	This study

yTA #	Short name	Genotype	pTA #	Use	Source
834	Arl3-GFP, 30Δ, h60, mCherry-Sed5	BY4741; *ARL3::GFP-HIS3MX6; naa30Δ::kanMX4; [pBEVY-U-hNAA60]; [p415Met25-mCherry-Sed5]*	286, 340	Fig. 5A	This study
1014	Arl3-GFP, 30Δ, $h60_{1\text{-}184}$, mCherry-Sed5	BY4741; *ARL3::GFP-HIS3MX6; naa30Δ::kanMX4; [pBEVY-U-hNAA60*$_{1\text{-}184}$*]; [p415Met25-mCherry-Sed5]*	340, 378	Fig. 5A	This study
1001	h60-EGFP	BY4741; *[pBEVY-U-hNAA60-EGFP]*	452	Fig. 5B	This study
1006	$h60_{1\text{-}182}$-EGFP	BY4742; *[pBEVY-U-hNAA60*$_{1\text{-}182}$*-EGFP]*	453	Fig. 5B	This study
639	Arl3-GFP, pBEVY-L	BY4741; *ARL3::GFP-HIS3MX6; [pBEVY-L]*	224	SG	This study
683	Arl3-GFP, 30Δ35Δ	BY4741; *ARL3::GFP-HIS3MX6; naa30Δ::kanMX4; naa35Δ::natNT2*		SG	This study
767	Arl3-GFP, 35Δ38Δ	BY4741; *ARL3::GFP-HIS3MX6; naa35Δ::kanMX4; naa38Δ::hphNT1*		SG	This study
748	Arl3-GFP, NatCΔ	BY4741; *ARL3::GFP-HIS3MX6; naa30Δ::kanMX4; naa35Δ::natNT2; naa38Δ::hphNT1*		SG	This study
686	Arl3-GFP, 30Δ35Δ, pBEVY-U	BY4741; *ARL3::GFP-HIS3MX6; naa30Δ::kanMX4; naa35Δ::natNT2; [pBEVY-U]*	53	SG	This study
688	Arl3-GFP, 30Δ35Δ, h30	BY4741; *ARL3::GFP-HIS3MX6; naa30Δ::kanMX4; naa35Δ::natNT2; [pBEVY-U-HA-hNAA30]*	231	SG	This study

Abbreviations: 30/35/38Δ; naa30/35/38Δ; h30/35/50/60; hNaa30/35/50/60; n.t., not tagged; p, plasmid; SG, strain generation; yTA#, internal yeast strain number; pTA#, internal plasmid number; TA, Thomas Arnesen lab; yTA, internal yeast strain number.

Table S2. The plasmids of this study.

pTA #	Name	Source
53	pBEVY-U	[2]
75	pcDNA3.1-*hNAA60-V5-His*	[3]
185	pCMV6-AC-*NAA35-GFP*	OriGene
224	pBEVY-L	[2]
226	pBEVY-L-*hNAA35-FLAG*	This study
229	pFA6a-*natNT2*	EUROSCARF
231	pBEVY-U-*HA-hNAA30*	This study
250	pFA6a-*hphNT1*	EUROSCARF
285	pBEVY-U-*hNAA50*	[4]
286	pBEVY-U-*hNAA60*	[3]
378	pBEVY-U-$hNAA60_{1\text{-}184}$	This study
321	316-*Sec7-mRFP*	RIKEN BRC DNA BANK
340	p415*Met25-mCherry-Sed5*	[5]
342	pBEVY-L-*hNAA35*	This study
407	p*hNAA60-EGFP-N1*	This study
439	p$hNAA60_{1\text{-}182}$*-EGFP-N1*	This study
452	pBEVY-U-*hNAA60-EGFP*	This study
453	pBEVY-U-$hNAA60_{1\text{-}182}$*-EGFP*	This study

Abbreviations: pTA#, internal plasmid number; TA, Thomas Arnesen lab

Table S3. Primers used for amplification of yeast and human genes.

**oyTA #**	**Sequence (5´ to 3´)**	**Use**	**Source**
49	CATGAAGAACAAAGTTTCAC	Amplify *kanMX4* with y*NAA30* flanks from genomic DNA of yTA44	[1]
50	CCTCTTTTCTCTACTGCC		
211	CTGTGCTAGTGTAGAGCAACTTTGATAATAGCTGGTACGTTTCCGAGAATGGAAGTAGACAGTATATTAGGCGGATCCCCGGGTTAATTAA	Amplify *natNT2* with y*NAA35* flanks from pTA229	This study
212	CCTTTATAATAATTAAATAGCGGGTATATAATGCTAAATATATTTTGTACAGTTAGGGTAAATTCCAGATTATTTATAGCGGGAATTCGAGCTCGATTACAA		
148	CTCCCACTTTAAAGTAAATCCGGAGATTCCC	Amplify *natNT2* with y*NAA35* flanks from genomic DNA of yTA683	This study
149	GGGGGAACGCACTGCATTTTACC		
244	GGGTGAAGATAGTCTTCAACAAACTTAGTAAGGGGCACCAAAGCGTCATGCGTACGCTGCAGGTCGAC	Amplify *hphNT1* with y*NAA38* flanks from pTA250	This study
245	GCCAAGCAATTGTTACTATGGAAACTATTAGAACCATATGCGCTAATCGATGAATTCGAGCTCG		
51	CTACCAAGAAACCGGGTAGC	PCR screen for *naa30*Δ	[1]
52	GAAACTTGCTTTATTATCTCTC		
146	CGTAGCTTCCCCAACTTCACCAG	PCR screen for *naa35*Δ	This study
147	CGAACTGTAACTCAAAGAAGCCTTGAAATTTACC		
9	CTCGCGAAGTGCATGAGTTCTGAC	PCR screen for *naa38*Δ	This study
11	GTTCAGTCCAGTTCATATTCG		
**oTA #**	**Sequence (5´ to 3´)**	**Use**	**Source**
99	CATTCTAGACTCGAGACCATGGTTATGAAAGCTTCTG	Amplify *hNAA35-FLAG* from pTA185	This study
98	CATCTGCAGTCACTTGTCATCGTCGTCCTTGTAATCAACAAGTTTCACAACAGG		
420	GTCGTCCTTGTAATCTCAAACAAGTTTCACAAC	Mutagenesis of pTA226, generating pTA342	This study
490	CCTATGTCCTCTACATCAACGGCGGCTGACCTCCCTGGACGATTTTGG	Mutagenesis of pTA286, generating pTA378	This study
17	GCGGGCTAGCATGACAGAGGTGGTGCCATCC	Amplify *hNAA60*$_{1-182}$ from pTA75	This study
463	GGTACCGGGTTGATGTAGAGGACATAGG		
18	GCGGGGTACCCCCATGGTCCGGCTGTACTCG	Amplify *hNAA60* from	This study

		pTA75 (together with oTA17)	
528	GATCATTCTAGAGAGAATGACAGAGGTGGTGC	Amplify *hNAA60-EGFP* from pTA407 and *hNAA60*$_{1-182}$*-EGFP* from pTA439	This study
529	GATCATGTCGACTTACTTGTACAGCTCGTCC		

Abbreviations: oTA, internal oligo number; oyTA, internal yeast oligo number; pTA#, internal plasmid number; TA, Thomas Arnesen lab

References Supplementary Tables

1 Aksnes, H., Osberg, C. & Arnesen, T. N-terminal acetylation by NatC is not a general determinant for substrate subcellular localization in Saccharomyces cerevisiae. *PLoS One* **8**, e61012,(2013).

2 Miller, C. A., Martinat, M. A. & Hyman, L. E. Assessment of aryl hydrocarbon receptor complex interactions using pBEVY plasmids: expression vectors with bi-directional promoters for use in Saccharomyces cerevisiae. *Nucleic Acids Research* **26**, 3577-3583,(1998).

3 Van Damme, P. *et al.* NatF contributes to an evolutionary shift in protein N-terminal acetylation and is important for normal chromosome segregation. *PLoS Genet* **7**, e1002169,(2011).

4 Van Damme, P., Hole, K., Gevaert, K. & Arnesen, T. N-terminal acetylome analysis reveals the specificity of Naa50 (Nat5) and suggests a kinetic competition between N-terminal acetyltransferases and methionine aminopeptidases. *Proteomics* **15**, 2436-2446,(2015).

5 Powis, K. *et al.* Get3 is a holdase chaperone and moves to deposition sites for aggregated proteins when membrane targeting is blocked. *Journal of Cell Science* **126**, 473-483,(2013).
